# Protein Loop Modeling via the Discretizable Distance Geometry Problem with Hydrogen-Based NMR Constraints

**DOI:** 10.1021/acsomega.5c06422

**Published:** 2026-02-10

**Authors:** Rômulo S. Marques, Michael Souza, Carlile Lavor

**Affiliations:** † Instituto de Matemática, Estatística e Computação Científica (IMECC - Unicamp), 28132 Universidade Estadual de Campinas, Campinas 13083-859, Brazil; ‡ Departamento de Estatística e Matemática Aplicada, Centro de Ciências, 28121 Universidade Federal do Ceará, Fortaleza 60020-181, Brazil

## Abstract

Protein loop modeling remains a fundamental challenge in computational biology due to the inherent flexibility of loops and their critical role in biological functions. In this work, we employ a discrete distance geometry formulation, efficiently solved using the Branch-and-Prune algorithm, with a key innovation being the incorporation of hydrogen atoms into the model. Hydrogen atoms bonded to *N* and *C*_α_ in the protein backbone introduce additional geometric constraints, and their inclusion is particularly justified in the context of nuclear magnetic resonance (NMR) experiments, where short-range hydrogen–hydrogen distances can be detected and provide valuable structural information. By integrating these experimentally accessible constraints into the modeling process, we refine the representation of protein conformations. Computational experiments demonstrate that incorporating hydrogen atoms reduces the conformational space, leading to a more constrained and biologically realistic model. Comparisons with hydrogen-free formulations confirm that our approach improves agreement with known protein structures, further highlighting the relevance of distance geometry methods in structural refinement.

## Graphical abstract

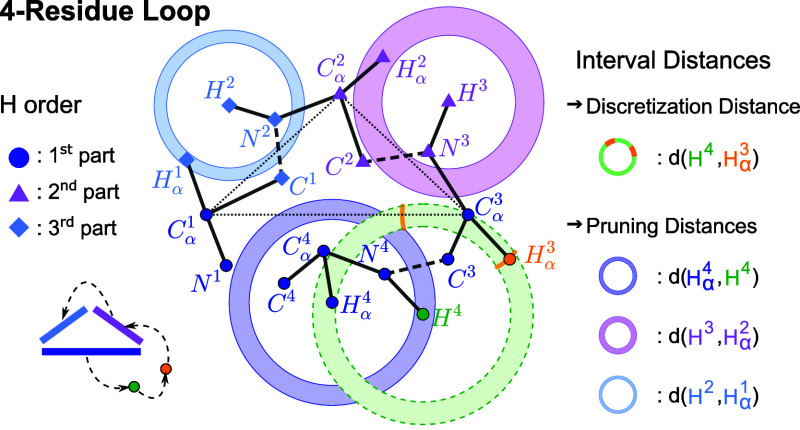

## Introduction

Among many important problems in computational biology related to the calculation of the three-dimensional structure of proteins, the *Tripeptide Loop Closure Problem* (TLCP) and its generalizations hold particular significance.^1–3^ While secondary structures such as α-helices and β-sheets exhibit relatively stable configurations, loops in protein backbones are often highly dynamic. These flexible fragments not only connect secondary structures but also play essential roles in processes such as signal transduction, protein–ligand binding, and protein–protein interactions.

The TLCP is defined as the problem of determining the ensemble of possible three-dimensional backbone structures for a three-residue segment of a protein, given by the atoms *N* ^*i*–2^, *C*_α_^*i*–2^, *C* ^*i*–2^, *N* ^*i*–1^, *C*_α_^*i*–1^, *C* ^*i*–1^, *N* ^*i*^, *C*_α_^*i*^, *C* ^*i*^, such that (see Figure 1):

- The positions of the atoms *C*_α_^*i*–2^, *C*_α_^*i*–1^, *C*_α_^*i*^ (as well as the distances among them) are known;
- The bond lengths and angles in the segment are fixed and also known;
- The sets {*C*_α_^*i*–2^, *C* ^*i*–2^, *N* ^*i*–1^, *C*_α_^*i*–1^}, {*C*_α_^*i*–1^, *C* ^*i*–1^, *N* ^*i*^, *C*_α_^*i*^}, and {*C*_α_^*i*^, *C* ^*i*^, *N* ^*i*–2^, *C*_α_^*i*–2^} are treated as rigid bodies.

**Figure 1:**
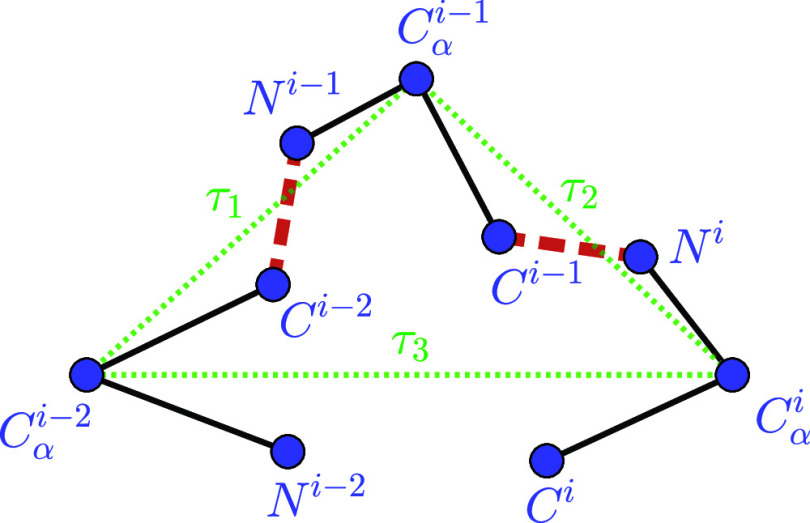
Backbone of a tripeptide loop (the dashed red lines represent the peptide bonds and the green dotted lines τ_1_, τ_2_, and τ_3_ indicate the virtual axes connecting *C*_α_^*i*–2^–*C*_α_^*i*–1^, *C*_α_^*i*–1^–*C*_α_^*i*^, and *C*_α_^*i*^–*C*_α_^*i*–2^, respectively).

The three atomic sets in the third condition form rigid bodies, since all pairwise intrabody distances are known (note that the atoms in each set lie on the same peptide plane).^4,5^ This rigid-body assumption is standard in TLCP formulations: it fixes the internal geometry of each body via exact intrabody distances derived from covalent bond lengths and bond angles, so that the loop motion is fully parametrized by three rotation angles (τ_1_, τ_2_, τ_3_ ∈ [0, 2π]) about the virtual axes connecting the fixed *C*_α_ atoms, namely *C*_α_^*i*–2^–*C*_α_^*i*–1^, *C*_α_^*i*–1^–*C*_α_^*i*^, and *C*_α_^*i*^–*C*_α_^*i*–2^ (see Figure 1). Accordingly, no additional degrees of freedom are introduced within a body, and the conformational variability addressed in the problem is entirely captured by τ_1_, τ_2_, and τ_3_.

Over the years, various mathematical formulations and algorithms have been developed to address the TLCP and its generalizations. Early studies modeled the problem using transcendental equations,^4^ while subsequent works used polynomial equations.^6^ More recently, the authors of ref 7 solved a relaxed version of the TLCP in which the bond *C*_α_^3^–*C* ^3^ can move freely in a 5-dimensional configuration space.

The authors of refs 8 and 9 attempt to bridge the gap between the robotics and molecular modeling communities by considering a more general version of the TLCP, formulated in terms of a system of Cayley-Menger determinants.^10,11^

The TLCP describes the simplest case of a loop closure problem, since it has the smallest possible number of amino acid residues. In a generalized version of the problem, the *Loop Closure Problem* (LCP), there are still three rigid bodies and the positions of the three *C*_α_ atoms that define them are still known. However, there may be more than four atoms in each rigid body.^12,13^

Throughout this manuscript we adopt the classical LCP setting, in which the positions of the three *C*_α_ atoms defining the rigid bodies are assumed to be known and are used to anchor the loop closure task. This assumption is standard in TLCP/LCP formulations and yields a well-posed subproblem by removing the indeterminacy due to global rigid motions. In addition, we treat each body as rigid by construction; if intrabody flexibility is desired, backbone dihedral angles within each body can be sampled in a separate upstream step (e.g., from Ramachandran distributions), the corresponding local geometry reconstructed, and the loop–closure problem solved for each sampled configuration.

In practical prediction scenarios and in NMR-driven modeling, however, loop boundaries may also be mobile and only partially determined. Modeling such boundary mobility naturally leads to more general formulations, such as the Molecular Distance Geometry Problem (MDGP),^14^ in which boundary atoms are treated as additional variables constrained by (possibly sparse and uncertain) distance information. Addressing this coupled setting is beyond the scope of the present study; here, we focus on the classical LCP as a principled and widely studied special case, and show how it can be tackled within a distance-geometry framework while incorporating hydrogen-based NMR-type restraints.

Figure 2 illustrates a six-residue loop, which, for simplicity, is assumed to begin at the first amino acid of the protein. The three rigid bodies in this case are defined by the sets {*C*_α_^1^, *C* ^1^, *N* ^2^, *C*_α_^2^, *C*_2_, *N* ^3^, *C*_α_^3^}, {*C*_α_^3^, *C* ^3^, *N* ^4^, *C*_α_^4^, *C*_4_, *N* ^5^, *C*_α_^5^}, and {*C*_α_^5^, *C* ^5^, *N* ^6^, *C*_α_^6^, *C*_6_, *N* ^1^, *C*_α_^1^}.

**Figure 2:**
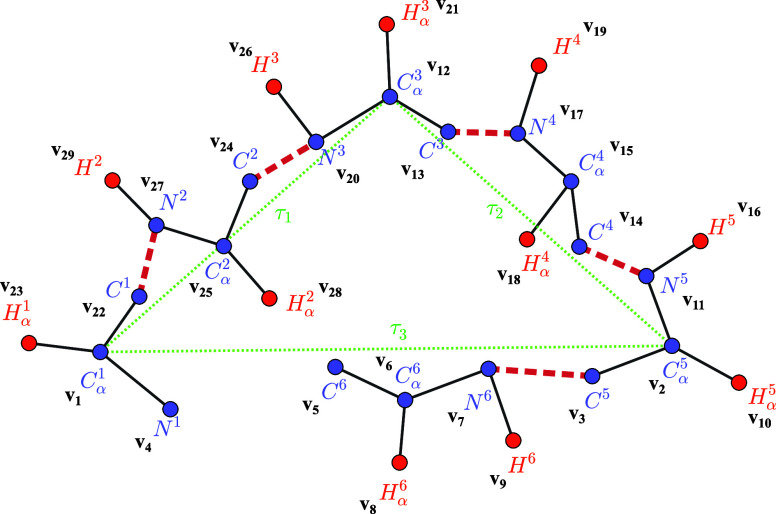
Backbone with hydrogens of a six-residues protein loop (*H*-order in black). The dashed red lines represent the peptide bonds and the green dotted lines τ_1_, τ_2_, and τ_3_ indicate the virtual axes connecting the three *C*_α_ atoms whose positions are known a priori (*C*_α_^1^, *C*_α_^3^, and *C*_α_^5^). Each atom is labeled with the symbol of its associated vertex, following the *H*-order. For example, the vertex associated with atom *C* ^6^ is *v*_5_, meaning it occupies the fifth position in the *H*-order.

In this work, we extend the distance geometry approach proposed in ref 13 to solve the LCP by incorporating hydrogen atoms bonded to the *N* and *C*_α_ atoms of the protein backbone. These hydrogen atoms introduce additional geometric constraints that enhance the accuracy of structural modeling, particularly in methods relying on Nuclear Magnetic Resonance (NMR) data, where short-range hydrogen–hydrogen distances provide valuable information.^15,16^

Our computational experiments demonstrate that the inclusion of hydrogen atoms leads to a more constrained and realistic conformational space, improving the robustness of distance-based modeling. Comparisons with models that exclude hydrogen atoms reveal that our approach achieves better consistency with known protein structures. These results highlight the potential of distance geometry methods in refining structural models and advancing our understanding of protein function.

## Discretizable Distance Geometry Problem

As in ref 13, we also model the LCP using the Discretizable Distance Geometry Problem (DDGP),^17,18^ which is defined in $R^{3}$ as follows.

**Definition 1 (DDGP)** *Consider a simple undirected graph G* = (*V*, *E*, *d*), *whose edges are weighted by d*: *E* → (0, *∞*), *and a total vertex order, denoted by v*_1_, ···, *v*_*n*_, *such that*

1. *For v*_1_, *v*_2_, *v*_3_ ∈ *V*, *there exist* $x_{1},x_{2},x_{3}\in R^{3}$ *satisfying* ([Disp-formula eq1]),
2. *For each i* ≥ 4, *there exist at least three predecessor vertices v*_*j*_,*v*_*k*_,*v*_*l*_ (*j* < *k* < *l*), *such that*
$$\{v_{j},v_{i}\},\{v_{k},v_{i}\},\{v_{l},v_{i}\}\in E$$
  *and*
$$d_{j,k}+d_{k,l}>d_{j,l}$$

*Find a function* $x:V\to R^{3}$ *such that*

$$\forall \left\{v_{i},v_{j}\right\}\in E,∥x_{i}-x_{j}∥=d_{i,j}$$ 1

*where x*_*i*_ = *x*(*v*_*i*_), *x*_*j*_ = *x*(*v*_*j*_), *d*_*i*,*j*_ = *d*({*v*_*i*_, *v*_*j*_}), *and* ||*x*_*i*_ – *x*_*j*_|| *is the Euclidean distance between x*_*i*_ *and x*_*j*_.

In the context of 3D protein structure calculations, the vertices of the DDGP graph represent atoms and the weighted edges correspond to pairs of atoms whose interatomic distances are known. The resolution of the problem consists of finding an embedding of the graph in $R^{3}$ , such that the computed distances between vertex positions match the weights of the corresponding edges.^14,19^

As previously mentioned in ref 13, the geometric properties of proteins and the *rigid geometry hypothesis*,^20,21^ which assumes that bond lengths and angles are fixed, enable the associated DDGP to be solved iteratively using the Branch-and-Prune (BP) method.^22–24^

A key aspect of this approach is the DDGP order,^25,26^ which structures the solution space as a binary tree. The construction of this tree relies on an initial assignment of fixed positions for the first three vertices, *v*_1_, *v*_2_, *v*_3_ (see the DDGP definition), which removes the ambiguity due to global rigid transformations, such as rotations and translations.^14^

Following this ordering, the position of each subsequent vertex is determined iteratively. Specifically, the placement of *v*_4_ is constrained by the known distances *d*_1,4_, *d*_2,4_, *d*_3,4_, which define three spheres centered at *v*_1_, *v*_2_, and *v*_3_, respectively. The intersection of these spheres yields at most two possible positions for *v*_4_, provided that the centers are noncollinear, a condition ensured by the strictness of the triangle inequality.^27^

This geometric framework forms the core of the BP algorithm, which systematically explores the solution space by iteratively determining the positions of subsequent vertices based on the intersection of spheres defined by known distances. By leveraging this combinatorial structure, the discrete formulation significantly reduces computational cost compared to continuous approaches.^28^

For vertices *v*_*i*_ with *i* ≥ 5, the BP algorithm extends this framework by incorporating possible additional distance constraints, ensuring that each new vertex is placed based on previously determined positions. Each new vertex *v*_*i*_ is positioned at the intersection of three spheres centered at the position of its predecessor vertices *v*_*j*_, *v*_*k*_, *v*_*l*_ (the set *E*_*d*_ of these edges is called the *discretization edge set*), with radii given by the corresponding distances. When an extra distance constraint {*v*_*r*_, *v*_*i*_} ∈ *E* (*r* < *i* – 3) is available (the set *E*_*p*_ of these edges is called the *pruning edge set*), it further refines the possible positions by introducing an additional sphere. If the centers of these four spheres are not coplanar and the intersection is nonempty, a unique position for *v*_*i*_ is determined. However, if the intersection is empty, the algorithm backtracks to explore alternative positions for the previous vertex. This process continues until a complete path from the root to a leaf is found, ensuring that all vertex positions satisfy the given distance constraints.

As a result, whenever a solution to the DDGP exists, the BP algorithm always finds a solution. Moreover, it can further explore the entire search tree to enumerate all possible solutions. In contrast, continuous methods are fundamentally limited to obtaining at most one solution, or none at all, since they may converge to local minima.^22,28^

## Integrating Hydrogen Atoms in Loop Modeling

In the backbone of a protein molecule, hydrogen atoms are bonded to both the *N* and *C*_α_ atoms (see Figure 2). When these hydrogen atoms are sufficiently close, their interatomic distances can be detected through NMR experiments, providing valuable geometric constraints that can be directly incorporated into distance-based modeling approaches.

To systematically integrate these NMR-based constraints, we present a DDGP order that explicitly accounts for hydrogen atoms (we call it *H*-order). This ordering is constructed using a six-residue instance of the problem, serving as a representative case that naturally extends to larger instances while preserving the combinatorial structure of the DDGP. This formulation enables a direct comparison with the DDGP order that excludes hydrogen atoms, as presented in ref 13.

For the six-residue instance considered in Figure 2, and assuming the vertex set *V* = {*v*_1_, *v*_2_, ···, *v*_29_}, we define below the set of edges *E*_*h*_ known a priori of the associated DDGP graph (this set includes both discretization edges and selected pruning edges):

$$\begin{array}{llll} & E_{h} & =\{ & \\ & & & \{v_{1},v_{2}\},\{v_{1},v_{3}\},\{v_{1},v_{4}\},\{v_{1},v_{5}\},\{v_{1},v_{6}\},\{v_{1},v_{7}\},\{v_{1},v_{12}\},\{v_{1},v_{20}\},\{v_{1},v_{22}\},\{v_{1},v_{23}\},\{v_{1},v_{24}\},\{v_{1},v_{25}\},\{v_{1},v_{27}\},\\ & & & \{v_{2},v_{3}\},\{v_{2},v_{4}\},\{v_{2},v_{5}\},\{v_{2},v_{6}\},\{v_{2},v_{7}\},\{v_{2},v_{10}\},\{v_{2},v_{11}\},\{v_{2},v_{12}\},\{v_{2},v_{13}\},\{v_{2},v_{14}\},\{v_{2},v_{15}\},\{v_{2},v_{16}\},\{v_{2},v_{17}\},\\ & & & \{v_{3},v_{4}\},\{v_{3},v_{5}\},\{v_{3},v_{6}\},\{v_{3},v_{7}\},\{v_{3},v_{9}\},\{v_{3},v_{10}\},\{v_{3},v_{11}\},\\ & & & \{v_{4},v_{5}\},\{v_{4},v_{6}\},\{v_{4},v_{7}\},\\ & & & \{v_{5},v_{6}\},\{v_{5},v_{7}\},\{v_{5},v_{8}\},\\ & & & \{v_{6},v_{7}\},\{v_{6},v_{8}\},\{v_{6},v_{9}\},\\ & & & \{v_{7},v_{8}\},\{v_{7},v_{9}\},\\ & & & \{v_{9},v_{10}\},\{v_{10},v_{11}\},\\ & & & \{v_{11},v_{12}\},\{v_{11},v_{13}\},\{v_{11},v_{14}\},\{v_{11},v_{15}\},\{v_{11},v_{16}\},\{v_{11},v_{17}\},\\ & & & \{v_{12},v_{13}\},\{v_{12},v_{14}\},\{v_{12},v_{15}\},\{v_{12},v_{17}\},\{v_{12},v_{20}\},\{v_{12},v_{21}\},\{v_{12},v_{22}\},\{v_{12},v_{24}\},\{v_{12},v_{25}\},\{v_{12},v_{26}\},\{v_{12},v_{27}\},\\ & & & \{v_{13},v_{14}\},\{v_{13},v_{15}\},\{v_{13},v_{17}\},\{v_{13},v_{19}\},\{v_{13},v_{20}\},\{v_{13},v_{21}\},\\ & & & \{v_{14},v_{15}\},\{v_{14},v_{16}\},\{v_{14},v_{17}\},\{v_{14},v_{18}\},\\ & & & \{v_{15},v_{17}\},\{v_{15},v_{18}\},\{v_{15},v_{19}\},\\ & & & \{v_{17},v_{18}\},\{v_{17},v_{19}\},\\ & & & \{v_{20},v_{21}\},\{v_{20},v_{22}\},\{v_{21},v_{24}\},\{v_{20},v_{25}\},\{v_{20},v_{26}\},\{v_{20},v_{27}\},\\ & & & \{v_{22},v_{23}\},\{v_{22},v_{24}\},\{v_{22},v_{25}\},\{v_{22},v_{27}\},\{v_{22},v_{29}\},\\ & & & \{v_{24},v_{25}\},\{v_{24},v_{26}\},\{v_{24},v_{27}\},\{v_{24},v_{28}\},\\ & & & \{v_{25},v_{27}\},\{v_{25},v_{28}\},\{v_{25},v_{29}\},\\ & & & \left\{v_{27},v_{28}\},\{v_{27},v_{29}\right\}\\ & & \} & \end{array}$$

where the predecessor sets are given by

$$\begin{array}{lll} & P_{v_{4}}(N^{1}) & =\{C_{\alpha }^{1},C_{\alpha }^{5},C^{5}\}=\{v_{1}^{b},v_{2}^{r},v_{3}^{r}\},\\ & P_{v_{5}}(C^{6}) & =\{C_{\alpha }^{1},C_{\alpha }^{5},C^{5},N^{1}=\{v_{1}^{r},v_{2}^{r},v_{3}^{r},v_{4}^{r}\},\\ & P_{v_{6}}(C_{\alpha }^{6}) & =\{C_{\alpha }^{1},C_{\alpha }^{5},C^{5},N^{1},C^{6}\}\\ & & =\{v_{1}^{r},v_{2}^{r},v_{3}^{b},v_{4}^{r},v_{5}^{b}\},\\ & P_{v_{7}}(N^{6}) & =\{C_{\alpha }^{1},C_{\alpha }^{5},C^{5},N^{1},C^{6},C_{\alpha }^{6}\}\\ & & =\{v_{1}^{r},v_{2}^{b},v_{3}^{b},v_{4}^{r},v_{5}^{b},v_{6}^{b}\},\\ & P_{v_{8}}(H_{\alpha }^{6}) & =\{C^{6},C_{\alpha }^{6},N^{1}\}=\{v_{5}^{b},v_{6}^{b},v_{7}^{b}\},\\ & P_{v_{9}}(H^{6}) & =\{C^{5},C_{\alpha }^{6},N^{6}\}=\{v_{3}^{b},v_{6}^{b},v_{7}^{b}\},\\ & P_{v_{10}}(H_{\alpha }^{5}) & =\{C_{\alpha }^{5},C^{5},H^{6}\}=\{v_{2}^{b},v_{3}^{b},v_{9}^{*}\},\\ & P_{v_{11}}(N^{5}) & =\{C_{\alpha }^{5},C^{5},H_{\alpha }^{5}\}=\{v_{2}^{b},v_{3}^{b},v_{10}^{b}\},\\ & P_{v_{12}}(C_{\alpha }^{3}) & =\{C_{\alpha }^{1},C_{\alpha }^{5},N^{1},N^{5}\}=\{v_{1}^{r},v_{2}^{r},v_{4}^{r},v_{11}^{r}\},\\ & P_{v_{13}}(C^{3}) & =\{C_{\alpha }^{5},N^{5},C_{\alpha }^{3}\}=\{v_{2}^{r},v_{11}^{r},v_{12}^{b}\},\\ & P_{v_{14}}(C^{4}) & =\{C_{\alpha }^{5},N^{5},C_{\alpha }^{3},C^{3}\}=\{v_{2}^{b},v_{11}^{b},v_{12}^{r},v_{13}^{r}\},\\ & P_{v_{15}}(C_{\alpha }^{4}) & =\{C_{\alpha }^{5},N^{5},C_{\alpha }^{3},C^{3},C^{4}\}\\ & & =\{v_{2}^{r},v_{11}^{b},v_{12}^{r},v_{13}^{b},v_{14}^{b}\},\\ & P_{v_{16}}(H^{5}) & =\{C_{\alpha }^{5},N^{5},C^{4}\}=\{v_{2}^{b},v_{11}^{b},v_{14}^{b}\},\\ & P_{v_{17}}(N^{4}) & =\{C_{\alpha }^{5},N^{5},C_{\alpha }^{3},C^{3},C^{4},C_{\alpha }^{4}\}\\ & & =\{v_{2}^{r},v_{11}^{r},v_{12}^{b},v_{13}^{b},v_{14}^{b},v_{15}^{b}\},\\ & P_{v_{18}}(H_{\alpha }^{4}) & =\{C^{4},C_{\alpha }^{4},N^{4}\}=\{v_{14}^{b},v_{15}^{b},v_{17}^{b}\},\\ & P_{v_{19}}(H^{4}) & =\{C^{3},C_{\alpha }^{4},N^{4}\}=\{v_{13}^{b},v_{15}^{b},v_{17}^{b}\},\\ & P_{v_{20}}(N^{3}) & =\{C_{\alpha }^{1},N^{1},C_{\alpha }^{3},C^{3}\}=\{v_{1}^{r},v_{4}^{r},v_{12}^{b},v_{13}^{b}\},\\ & P_{v_{21}}(H_{\alpha }^{3}) & =\{C_{\alpha }^{3},C^{3},N^{3}\}=\{v_{12}^{b},v_{13}^{b},v_{20}^{b}\},\\ & P_{v_{22}}(C^{1}) & =\{C_{\alpha }^{1},N^{1},C_{\alpha }^{3},N^{3}\}=\{v_{1}^{b},v_{4}^{b},v_{12}^{r},v_{20}^{r}\},\\ & P_{v_{23}}(H_{\alpha }^{1}) & =\{C_{\alpha }^{1},N^{1},C^{1}\}=\{v_{1}^{b},v_{4}^{b},v_{22}^{b}\},\\ & P_{v_{24}}(C^{2}) & =\{C_{\alpha }^{1},N^{1},C_{\alpha }^{3},N^{3}\}=\{v_{1}^{r},v_{4}^{r},v_{12}^{b},v_{20}^{b}\},\\ & P_{v_{25}}(C_{\alpha }^{2}) & =\{C_{\alpha }^{1},N^{1},N^{3},C^{2}\}=\{v_{1}^{r},v_{4}^{r},v_{20}^{b},v_{24}^{b}\},\\ & P_{v_{26}}(H^{3}) & =\{C_{\alpha }^{3},N^{3},C^{2}\}=\{v_{12}^{b},v_{20}^{b},v_{24}^{b}\},\\ & P_{v_{27}}(N^{2}) & =\{C_{\alpha }^{1},N^{1},N^{3},C^{1},C^{2},C_{\alpha }^{2}\}\\ & & =\{v_{1}^{b},v_{4}^{r},v_{20}^{r},v_{22}^{b},v_{24}^{b},v_{25}^{b}\},\\ & P_{v_{28}}(H_{\alpha }^{2}) & =\{C^{2},C_{\alpha }^{2},N^{2}\}=\{v_{24}^{b},v_{25}^{b},v_{27}^{b}\},\\ & P_{v_{29}}(H^{2}) & =\{C^{1},C_{\alpha }^{2},N^{2}\}=\{v_{22}^{b},v_{25}^{b},v_{27}^{b}\} \end{array}$$

To better understand this notation, let us examine the following three cases.

- *P*_*v*_5__(*C* ^6^) = {*C*_α_^1^, *C*_α_^5^, *C* ^5^, *N* ^1^} = {*v*_1_^*r*^, *v*_2_^*r*^, *v*_3_^*r*^, *v*_4_^*r*^}

The atom *C* ^6^ belongs to the sixth residue, corresponds to vertex *v*_5_ in the ordering, and has known distances to four preceding atoms (*C*_α_^1^, *C*_α_^5^, *C* ^5^, *N* ^1^), which are identified as vertices *v*_1_, *v*_2_, *v*_3_, *v*_4_, respectively. The symbol *r* indicates that these distance values are known because all the atoms belong to the same rigid body.

- *P*_*v*_10__(*H*_α_^5^) = {*C*_α_^5^, *C* ^5^, *H* ^6^} = {*v*_2_^*b*^, *v*_3_^*b*^, *v*_9_^*^}

The atom *H*_α_^5^ belongs to the fifth residue, corresponds to vertex *v*_10_ in the ordering, and has known distances to only three preceding atoms (the minimum required to establish a DDGP order), namely *C*_α_^5^, *C* ^5^, *H* ^6^, which are identified as vertices *v*_2_, *v*_3_, *v*_9_, respectively. The symbol *b* in *v*_2_^*b*^ indicates that the distance *d*(*v*_2_, *v*_10_) = *d*(*C*_α_^5^, *H*_α_^5^) is known because the atom *H*_α_^5^ is separated from *C*_α_^5^ by at most two covalent bonds. Note that although *H*_α_^5^ is directly bonded to *C*_α_^5^, it is two covalent bonds away from the atom *C* ^5^.

The symbol ∗ in *v*_9_^*^ indicates that the distance *d*(*v*_9_, *v*_10_) = *d*(*H* ^6^, *H*_α_^5^) is known under the assumption that these two hydrogen atoms are sufficiently close to be detected by NMR experiments.^29,30^ Due to the inherent inaccuracy of experimental data, this distance is considered an interval distance. In the worst-case scenario, if this distance is not detected, its minimum and maximum values can be estimated using the same approach as in ref 13.

When computing the position of a vertex based on two exact distances and one interval distance, like *v*_10_, the BP algorithm applies a discretization strategy: the interval is uniformly sampled into *K* values, where *K* is an input parameter specifying the number of samples. For each sampled value, a third sphere is constructed and intersected with the two spheres defined by the exact distances. Since this corresponds to the intersection of three spheres, the vertex is positioned using the same geometric procedure described in the previous section. Consequently, each of the *K* sampled values yields at most two candidate positions, potentially resulting in up to 2*K* candidates for the vertex. More details about how BP handles interval distances can be found in refs 13 and 31.

Among all distances associated with the set of discretization edges *E*_*d*_, *d*(*H* ^6^, *H*_α_^5^) is the only one that neither belongs to a rigid body nor is directly related to bond lengths or bond angles. Naturally, other distances detected by NMR, which will be used as pruning distances, will also be treated as interval distances.

- *P*_*v*_12__(*C*_α_^3^) = {*C*_α_^1^, *C*_α_^5^, *N* ^1^, *N* ^5^} = {*v*_1_^*r*^, *v*_2_^*r*^, *v*_4_^*r*^, *v*_11_^*r*^}

In this case, the atom *v*_12_ = *C*_α_^3^ belongs to two rigid bodies. The distances *d*(*v*_1_, *v*_12_) = *d*(*C*_α_^1^, *C*_α_^3^) and *d*(*v*_2_, *v*_12_) = *d*(*C*_α_^5^, *C*_α_^3^) are known because the atom pairs (*C*_α_^1^, *C*_α_^3^) and (*C*_α_^5^, *C*_α_^3^) lie within the same rigid body.

To provide a clearer understanding of the structure of the search space of the problem, Figure 3 presents a compact version of the BP tree for the six-residue loop problem. Subtrees that are not explicitly depicted are represented by blue triangles. Gray circles denote vertices that do not branch into two possible configurations, that is, they have at least four predecessors, such as *P*_*v*_5__(*C* ^6^). In contrast, green circles represent branching vertices. The vertex *v*_10_ = *H*_α_^5^, which is subject to the interval constraint *d*(*v*_9_, *v*_10_) = *d*(*H* ^6^, *H*_α_^5^), is illustrated as an arc because the intersection of two spheres and a spherical shell typically results in one or two circular arcs.^31,32^

**Figure 3:**
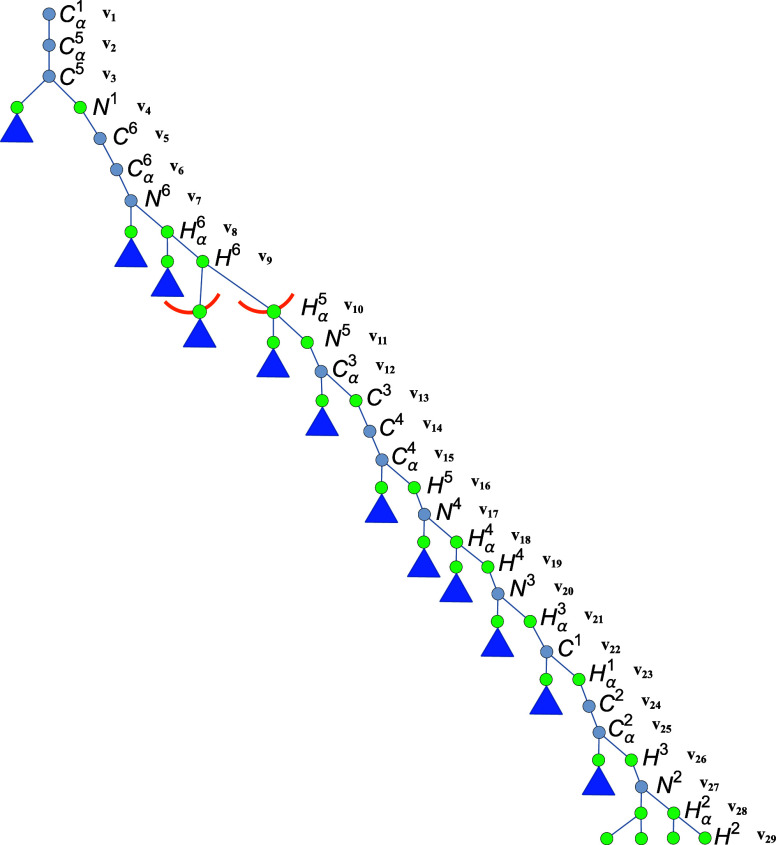
Compact representation of the BP tree for the six-residue loop problem that uses the *H*-order. Blue triangles indicate subtrees that are not fully shown. Gray circles represent vertices with a unique configuration, while green circles denote branching vertices. The vertex *v*_10_ = *H*_α_^5^, subject to the interval *d*(*v*_9_, *v*_10_) = *d*(*H* ^6^, *H*_α_^5^), is represented as an arc, indicating the range of possible positions consistent with the given distance interval.

At first glance, the definition of *E*_*h*_ may appear to be instance-dependent. However, it naturally extends to all instances, since the essential aspect of the construction is the use of carbon atoms with known positions, which is identical in every case. The only variation lies in the number of vertices within each rigid body, which increases according to the same underlying pattern.

## Discussion and Computational Results

The primary motivation for defining a new DDGP order in the context of protein loop modeling using available NMR data is based on the idea that incorporating hydrogen atoms can reduce the search space of the problem. This reduction is not arbitrary: it aims to eliminate only those conformations that violate hydrogen–hydrogen distance constraints. As a result, the remaining solutions would not be only fewer but also more consistent with experimental data, offering a more biologically meaningful ensemble of conformations.

In addition to introduce hydrogen atoms, which generate possible pruning edges (a feature absent from the ordering used in ref 13), the *H*-order exhibits another important difference compared to the one adopted in ref 13. The interval distance used as a discretization edge (only one in both orderings) has two properties that distinguish it from that of:^13^

1. It appears after the fourth vertex, whereas in ref 13 the interval distance is used exactly at their fourth vertex; in our case, this later placement reduces the size of the search space.
2. It can be derived from NMR measurements, and the smaller the associated interval, the smaller the resulting search space.

To verify that a reduction in the search space indeed occurs, we analyze the results obtained by the BP solver when applied to two DDGP orderings: (a) the *H*-order, which includes all hydrogen atoms bonded to the backbone; (b) a “reduced” *H*-order, referred to as the *H̅* order, which contains only the two hydrogens involved in the unique interval discretization distance (see distance *d*(*v*_9_, *v*_10_) = *d*(*H* ^6^, *H*_α_^5^) in the previous section).

Note that, with the exception of a single nitrogen atom (see *P*_*v*_11__(*N* ^5^) in the previous section, where the predecessor sets for the *H*-order are defined), the coordinates of all other backbone atoms are computed using exact distances to neighboring atoms that also belong to the backbone, that is, they are not hydrogen atoms. On the other hand, the nitrogen *N* ^5^ and the hydrogens *H*_α_^5^, *H* ^6^ have the same predecessor sets in both the *H* and *H̅* orders. Consequently, removing from the *H*-order all hydrogens not associated with the interval discretization distance *d*(*v*_9_, *v*_10_) = *d*(*H* ^6^, *H*_α_^5^) results in an ordering *H̅* that still satisfies the DDGP requirements.

All algorithms were implemented in Python, building upon the source code provided in ref 13, and are publicly available at https://github.com/romulomarques/bpl. Computational experiments were conducted on a machine equipped with an Intel­(R) Core­(TM) i9-13900H CPU running at 2.60 GHz, 16 GB of RAM, and the Linux Ubuntu 22.04.5 LTS operating system.

### DDGP Instances

The tests were performed on the same PDB instances used in ref 13, which also addresses the problem using a distance geometry-based approach, but employs a DDGP order that excludes hydrogen atoms. In that work, the presented approach is compared with an algorithm proposed in ref 12, called CSJD, which is based on the calculation of roots of polynomial systems. The benchmark set comprises 29 loops, grouped by length into three categories of 4, 8, and 12 residues, selected from a data set of nonredundant X-ray crystallographic structures.

PDB files typically provide 3D coordinates for each atom, but most entries do not contain hydrogen atoms. Therefore, we have developed simple and consistent procedures to provide the coordinates of missing hydrogen atoms.

First, consider hydrogen atoms bonded to nitrogen atoms (*H* ^*i*^). These atoms belong to the so-called *peptide plane* (for example, in the case of residues *i* – 1 and *i*, the atoms within this plane are *C*_α_^*i*–1^, *C* ^*i*–1^, *N* ^*i*^, *C*_α_^*i*^, and *H* ^*i*^). As all interatomic distances within this plane can be considered known a priori, we reconstruct the position of each *H* ^*i*^ atom by computing the intersection of four spheres centered at *C*_α_^*i*–1^, *C* ^*i*–1^, *N* ^*i*^, and *C*_α_^*i*^ (whose coordinates are provided in the PDB), with radii corresponding to their distances to *H* ^*i*^ (taken from the values reported in refs 33 and 34).

To determine the coordinates of the hydrogen atoms bonded to the α carbon (*H*_α_^*i*^), we apply the same procedure, using spheres centered at *N* ^*i*^, *C*_α_^*i*^, *C* ^*i*^, and *C*_β_^*i*^, with radii corresponding to the distances to *H*_α_^*i*^ (derived from bond lengths and bond angles provided in ref 33).

Using the resulting 3D coordinates of *H* ^*i*^ and *H*_α_^*i*^ atoms, we generate the DDGP instances required for our computational experiments.

The interval distances associated with hydrogen atoms (including *H*_α_^5^ and *H* ^6^) are computed as follows. Given their positions (as reconstructed above), we evaluate their interatomic distance *d* (in Å). If *d* < 5, we define an interval of length δ, given by [*d* – τ, *d* + δ – τ], where τ is a random real number drawn uniformly from [0, δ]. The 5 Å threshold is chosen to simulate distance values typically obtained from NMR experiments.

Finally, for each DDGP instance, we generate four versions of interval distance constraints by selecting δ ∈ {0.2, 0.5, 1.0, 1.5}, and Tables 1–3 report only the most challenging case, corresponding to the largest interval length δ = 1.5 (see Figure 4 for results with smaller δ). Complete results for all δ values are available as described in the *Data and Software Availability* section.

**Table 1:** Summary of the Results Obtained by the (*BP*)_*H*_ Algorithm (Our Method Using *H*-order), Compared with the CSJD and BP Methods on *Four-Residue* Loop Instances^a^

loop	interval distance	CSJD (rmsd, sol)	BP (rmsd, sol)	(*BP*)_*H*_ (rmsd, sol)	tsecs	max_err
1dvjA(20,21,22)	[2.50, 4.00]	0.38 (4548)	0.00 (5394)	0.03 **(132)**	006.61	0.01
1dysA(47,48,49)	[1.78, 3.28]	0.37 (2234)	0.00 (952)	0.00 (6517)	057.41	0.01
1eguA(404,405,406)	[2.35, 3.85]	0.37 (170)	0.00 (544)	0.00 (1016)	156.06	0.01
1ejoA(74,75,76)	[2.84, 4.34]	0.21 (1564)	0.13 (288)	0.01 **(84)**	019.94	0.01
1i0hA(123,124,125)	[1.62, 3.12]	0.26 (342)	0.00 (516)	0.00 (3206)	083.51	0.01
1id0A(405,406,407)	[2.72, 4.22]	0.72 (528)	0.01 (70)	0.00 **(56)**	041.04	0.00
1qnrA(195,196,197)	[2.95, 4.45]	0.39 (1064)	0.01 (28)	0.01 (36)	007.01	0.01
1qopA(44,45,46)	[2.89, 4.39]	0.61 (4284)	0.02 (1980)	0.01 **(266)**	015.31	0.01
1tcaA(95,96,97)	[1.69, 3.19]	0.28 (418)	0.00 (956)	0.00 **(70)**	011.88	0.01
1thfD(121,122,123)	[2.91, 4.41]	0.36 (2958)	0.00 (756)	0.00 **(102)**	007.43	0.01

a The first column shows the PDB code and the three residues where the *C*_α_ atoms are fixed. The second column gives the interval used for the discretization edge. Columns 3 to 5 report the minimum RMSD values (in Å) and the number of solutions found (in parentheses) for the CSJD, BP, and (*BP*)_*H*_ methods. Columns 6 and 7 apply only to (*BP*)_*H*_, showing the total runtime (in seconds) and the maximum distance constraint violation (max_err). Bold entries highlight cases where (*BP*)_*H*_ found fewer solutions than both comparison methods. All results use 1000 samples from the interval in column two.

**Figure 4:**
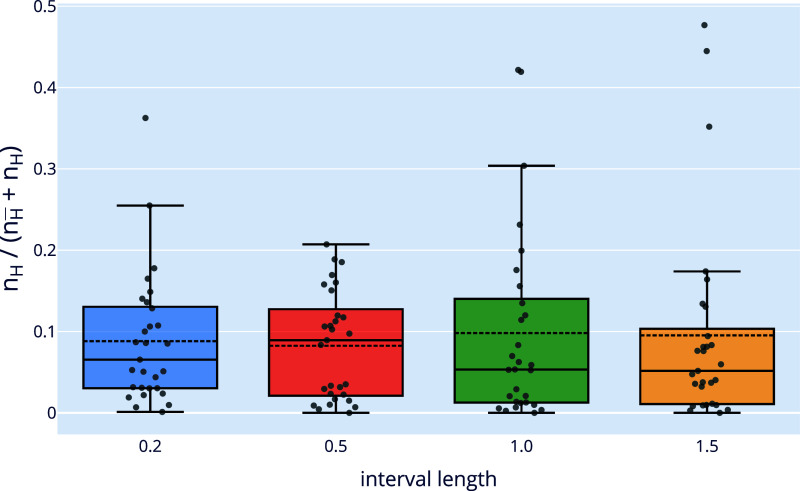
Boxplots of the ratio *n*_*H*_/(*n*_*H̅*_ + *n*_*H*_), where *n*_*H*_ and *n*_*H̅*_ denote the number of solutions found by the BP algorithm using the *H* and *H̅* DDGP orders, respectively. Each boxplot summarizes results over the full benchmark set of loop instances. The four experiments differ only in the interval length δ used to generate hydrogen–hydrogen interval constraints, set to 0.2, 0.5, 1.0, and 1.5 Å (*x*-axis).

### BP and CSJD Methods

Before comparing the *H* and *H̅* orders, we first present how the BP algorithm performs when using the *H*-order, denoted in the tables as (*BP*)_*H*_. This performance is compared with the results reported in.^13^ To simplify the comparison, and given that the main quality indicator is the number of solutions found, we focus on this quantity when comparing (*BP*)_*H*_ to the two methods evaluated in^13^ the BP algorithm with a different DDGP order (excluding hydrogens), denoted simply by BP, and the CSJD method, which is based on the calculation of polynomial roots.

At first glance, one might argue that the number of solutions obtained by (*BP*)_*H*_ is not directly comparable to those obtained by CSJD and BP, since hydrogen atoms are not considered in the latter two methods. To give the comparison a meaningful context, we assume that the problem is posed in an NMR environment, where available information on the distance between nearby hydrogen atoms is not used by the CSJD and BP methods. Ideally, the comparison would involve modified versions of CSJD and BP that incorporate NMR information. Since such versions are currently unavailable, we present the most informative comparison possible under this limitation. Tables 1–3 summarize the relevant results associated with loop segments of 4, 8, and 12 residues, respectively.

From this perspective, we may state that the distance geometry approach can effectively incorporate NMR-based information, yielding superior results (according to the above-mentioned criteria) when compared to the two other methods that ignore this information.

The first column in each table indicates the PDB protein ID concatenated with the chain that is being analyzed, along with the identification of the three residues for which the *C*_α_ atoms are fixed. For example, in the entry 1dvjA­(20,21,22) from Table 1, the PDB ID is “1dvj”, the chain is “A”, the loop begins at residue 20, and the fixed *C*_α_ atoms are located at residues 20, 21, and 22. In this particular table, each loop contains four residues, and since the fixed residues are consecutive, the loop naturally ends at residue 23. Accordingly, the first, second, and third rigid bodies include atoms from residues {20, 21}, {21, 22}, and {22, 23, 20}, respectively.

The second column gives the numerical range associated with the interval discretization edge computed for each loop (in the instance with 6 residues, this value is given by *d*_9,10_). For the CSJD, BP, and (*BP*)_*H*_ methods, the third, fourth, and fifth columns report the minimum root-mean-square deviation (RMSD) values (in Å) with respect to the reference PDB structures, together with the number of candidate loops found (in parentheses).For each method, the RMSD is calculated considering all the atoms available, that is, backbone atoms for the CJSD and BP methods, and backbone plus hydrogens for the (*BP*)_*H*_. When the RMSD value is nearly zero, it indicates that the predicted structure matches the PDB structure. The reported RMSD corresponds to the smallest value among all generated solutions for that instance; other feasible solutions generally differ geometrically and yield larger RMSD values, which are therefore not shown in the tables.

The next two columns refer exclusively to the (*BP*)_*H*_ method. The first reports the total computational time (in seconds) required to find all solutions. The last column gives the value of max_err, defined as

$$\text{max\_err}=\max_{\{i,j\}\in E}\{|∥x_{i}-x_{j}∥-d_{i,j}|\}$$ 2

Since the max_err values are nearly zero in all cases, it follows that every structure generated by the (*BP*)_*H*_ algorithm satisfies the distance constraints imposed by the problem.

In all three tables, for each loop instance, we uniformly sampled 1000 values from the interval specified in the second column.

Furthermore, in each table, we highlight in bold the cases where the number of solutions (*sol*) found by the (*BP*)_*H*_ method is smaller than those obtained by the CSJD and BP methods.

For example, in the second loop of Table 3 (which corresponds to the longest loops), the CSJD and BP methods find structures with RMSD values equal to 1.60 and 0.00, respectively, with 1802 and 610 candidate loops. In contrast, the (*BP*)_*H*_ algorithm finds a structure with an RMSD of 0.00 based on only 24 candidate loops.

Considering all 29 loops, the (*BP*)_*H*_ algorithm produces fewer solutions than the CSJD and BP methods in 24 cases, which represents 80% of the data set.

In Table 1 (loops of 4 residues), (*BP*)_*H*_ yields a lower number of solutions in 60% of the cases. In 5 out of these 6 instances, compared to the method yielding the fewest solutions between CSJD and BP, the (*BP*)_*H*_ algorithm achieves at least a 70% reduction in the number of solutions, reaching a 97% reduction in the remaining case.

To interpret the solution counts in Tables 1–3, note that the number of conformations returned by (*BP*)_*H*_ is driven not only by loop flexibility, but also by the amount of hydrogen-based pruning information available and by how early it can be applied in the search tree. In our setting, interhydrogen contacts below 5 Å yield interval restraints that can be exploited as pruning edges; for short loops (4 residues) the number of such contacts is typically smaller, which limits pruning and may leave more candidate conformations feasible. As the loop length increases (8 and 12 residues), the number of nearby hydrogen pairs generally increases, providing more pruning edges and leading to a stronger reduction in the number of feasible conformations (this effect remains instance-dependent, since the available contacts vary across segments).

Moreover, despite incorporating additional constraints, (*BP*)_*H*_ may still find more solutions than the conventional backbone-only BP in a few instances. This outcome can be attributed to the improved numerical stability provided by the *H*-ordering: it is well established that orderings that embed atoms in $R^{3}$ using shorter, local distances tend to accumulate less numerical error, whereas orderings that require embedding using longer distances may accumulate enough error to inadvertently discard solutions that are theoretically feasible.^18^ This difference is reflected in the interval distance that each method discretizes. In (*BP*)_*H*_, the discretized interval corresponds to a pair of hydrogens from consecutive residues and is therefore typically smaller than 5 Å (see column 2 of Tables 1–3). In contrast, under the ordering used in,^13^ the discretized interval distance can be substantially larger, and may exceed 11 Å in longer loops, which can amplify numerical effects.

In Table 2 (loops of 8 residues), (*BP*)_*H*_ outperforms the comparison methods in all cases (100%). For these 10 loops, compared to the best-performing method among CSJD and BP, (*BP*)_*H*_ reduces the number of solutions by at least 93%, and by more than 97% in 8 of the 10 cases.

**Table 2:** Summary of the Results Obtained by the (*BP*)_*H*_ Algorithm (Our Method Using *H*-order), Compared with the CSJD and BP Methods on *Eight-Residue* Loop Instances^a^

loop	interval distance	CSJD (rmsd, sol)	BP (rmsd, sol)	(*BP*)_*H*_ (rmsd, sol)	tsecs	max_err
1cruA(85,87,89)	[2.98, 4.48]	0.99 (2516)	0.02 (268)	0.02 **(2)**	079.50	0.01
1ctqA(144,146,148)	[2.77, 4.27]	0.96 (1754)	0.00 (476)	0.00 **(6)**	063.83	0.01
1d8wA(334,336,338)	[2.54, 4.04]	0.37 (1686)	0.00 (1568)	0.01 **(12)**	071.94	0.01
1ds1A(20,22,24)	[2.98, 4.48]	1.30 (3506)	0.01 (1222)	0.01 **(4)**	031.96	0.00
1gk8A(122,124,126)	[2.25, 3.75]	1.29 (2362)	0.00 (492)	0.00 **(6)**	029.19	0.01
1i0hA(145,147,149)	[2.98, 4.48]	0.36 (1452)	0.02 (32)	0.01 **(2)**	017.46	0.00
1ixhA(106,108,110)	[1.77, 3.27]	2.36 (4448)	0.01 (912)	0.00 **(20)**	026.14	0.01
1lamA(420,422,424)	[1.97, 3.47]	0.83 (2200)	0.00 (672)	0.01 **(32)**	554.20	0.01
1qopB(14,16,18)	[1.58, 3.08]	0.69 (3384)	0.00 (448)	1.70 **(8)**	177.88	0.01
3chbD(51,53,55)	[2.63, 4.13]	0.96 (1838)	0.02 (466)	0.01 **(4)**	026.31	0.01

a The first column shows the PDB code and the three residues where the *C*_α_ atoms are fixed. The second column gives the interval used for the discretization edge. Columns 3 to 5 report the minimum RMSD values (in Å) and the number of solutions found (in parentheses) for the CSJD, BP, and (*BP*)_*H*_ methods. Columns 6 and 7 apply only to (*BP*)_*H*_, showing the total runtime (in seconds) and the maximum distance constraint violation (max_err). Bold entries highlight cases where (*BP*)_*H*_ found fewer solutions than both comparison methods. All results use 1000 samples from the interval in column two.

In Table 3 (loops of 12 residues), (*BP*)_*H*_ shows improvement in 77% of the cases. Among the 9 instances, in 6 of them the number of solutions was reduced by at least 95% compared to the best result from CSJD and BP, and in the 1qopA case by 83%. For the 1ctqA instance, all backbone solutions obtained by (*BP*)_*H*_ violate at least one hydrogen–hydrogen distance. This outcome is likely due to the fact that, during the discretization of the interval distance between hydrogens, the number of sampled values within the interval (*K* = 1000) was insufficient for the method to identify a solution satisfying all constraints. Therefore, to obtain feasible solutions, it is necessary to increase the number of sampled points in the interval shown in the second column of the table.

**Table 3:** Summary of the Results Obtained by the (*BP*)_*H*_ Algorithm (Our Method Using *H*-order), Compared with the CSJD and BP Methods on *12-Residue* Loop Instances^a^

loop	interval distance	CSJD (rmsd, sol)	BP (rmsd, sol)	(*BP*)_*H*_ (rmsd, sol)	tsecs	max_err
1ctqA(26,29,32)	[1.56, 3.06]	1.86 (3968)	0.01 (694)			
1d4oA(88,91,94)	[2.92, 4.42]	1.60 (1802)	0.00 (610)	0.00 **(24)**	0024.42	0.01
1d8wA(46,49,52)	[2.15, 3.65]	2.94 (3906)	0.13 (614)	0.00 **(10)**	0082.15	0.01
1ds1A(282,285,288)	[2.23, 3.73]	3.10 (1162)	0.00 (24)	0.00 (158)	3514.08	0.01
1dysA(291,294,297)	[1.87, 3.37]	3.04 (2306)	0.02 (238)	0.05 **(10)**	0040.35	0.01
1eguA(508,511,514)	[2.85, 4.35]	2.82 (2106)	6.24 (734)	0.01 **(8)**	0218.69	0.01
1f74A(11,14,17)	[3.00, 4.50]	1.53 (3048)	0.26 (954)	0.13 **(4)**	0125.86	0.01
1q1wA(31,34,37)	[2.90, 4.40]	2.32 (4780)	0.08 (112)	0.04 **(4)**	0062.70	0.01
1qopA(178,181,184)	[2.88, 4.38]	2.18 (2014)	0.01 (472)	0.00 **(76)**	0771.84	0.01

a The first column shows the PDB code and the three residues where the *C*_α_ atoms are fixed. The second column gives the interval used for the discretization edge. Columns 3 to 5 report the minimum RMSD values (in Å) and the number of solutions found (in parentheses) for the CSJD, BP, and (*BP*)_*H*_ methods. Columns 6 and 7 apply only to (*BP*)_*H*_, showing the total runtime (in seconds) and the maximum distance constraint violation (max_err). Bold entries highlight cases where (*BP*)_*H*_ found fewer solutions than both comparison methods. All results use 1000 samples from the interval in column two.

Finally, to solve the 1ds1A instance in Table 3, (*BP*)_*H*_ required substantially more time than for the other loops. This can be due to two factors. First, this instance admits a considerably larger number of solutions that satisfy the hydrogen constraints compared with the other instances of the same length. Second, pruning likely occurs at deeper levels of the solution tree, leading to a more extensive exploration of the binary tree. In contrast, for the 1d4oA instance, which has a similar length, the problem was solved in only 24 s, probably because pruning takes place at higher levels of the tree, thereby significantly reducing the search space. It is worth noting that the number of nodes in the binary solution tree grows exponentially with the length of the instance.

Computational time is, of course, an important practical criterion for loop–closure methods, and for completeness we report the total running times of (*BP*)_*H*_ in Tables 1–3. As discussed above, the runtime is largely governed by the pruning pattern in the binary search tree and therefore need not increase monotonically with additional constraints (effective pruning can substantially reduce the number of explored nodes).

Importantly, the purpose of the present work is not to claim a general speed advantage over CSJD or the backbone-only BP variant, but to quantify how hydrogen-based constraints refine the solution set by discarding conformations that remain feasible under backbone-only geometry yet are structurally inconsistent. The reported times indicate that this refinement is achieved with a computational cost that remains practical for the loop-closure setting considered here.

Although CSJD is a highly efficient, specialized loop-closure solver, the distance-geometry-based pipeline considered here remains computationally practical for the instances in Tables 1–3 (see the reported runtimes). The main contribution of our formulation is therefore not speed per se, but improved solution quality and stronger discrimination as the loop length increases. In our benchmarks, CSJD achieves sub-Å fits for short loops (e.g., ∼0.2–0.3 Å for 4-residue cases), but its best RMSD values more frequently move to the multi-Å range for longer loops (typically ∼1–3 Å for 12-residue cases). In contrast, BP-based distance geometry still recovers near-native conformations with minimum RMSD values close to zero in most instances across loop lengths. Moreover, incorporating hydrogen–hydrogen interval restraints motivated by NMR data significantly reduces the number of conformations consistent with the distance information, yielding fewer—but structurally more constrained—candidate loops.

The RMSD values reported in Tables 1–3 correspond to the *minimum* RMSD among all solutions generated for each instance and method, and therefore they do not characterize the full distribution of conformations. In particular, even when the reported minimum RMSD is close to zero, other feasible solutions may deviate substantially from the reference PDB structure while still satisfying the distance constraints (see the complete tables with maximum/average RMSD values provided in the *Data and Software Availability* section). Importantly, the benefit of incorporating backbone-bonded hydrogens should not be interpreted primarily as an improvement in the minimum RMSD, but as a refinement of the feasible set: hydrogen–hydrogen interval restraints prune backbone conformations that remain feasible under backbone-only constraints yet are inconsistent with additional short-range contacts. As a result, (*BP*)_*H*_ typically retains fewer—but more structurally constrained—candidate loops, while maintaining very small distance-violation levels (cf. the max_err column). In this context, instances with larger minimum RMSD values (e.g., 1qopB) indicate conformations that differ from the specific deposited reference model, rather than geometrically invalid solutions.

### Comparing *H* and *H̅* Orders

Now, we investigate whether hydrogen–hydrogen distance constraints, particularly those obtainable through NMR experiments, can be effectively exploited to eliminate infeasible loop conformations within the DDGP framework.

In the DDGP with ordering *H*, all distances between hydrogen pairs separated by up to 5 Å are known. Consequently, the number of available distances depends on how many nearby hydrogen pairs each instance contains. Among these distances, only one is used for discretization to place an atom in $R^{3}$ , while the others serve for pruning. In contrast, in the DDGP with ordering *H̅*, there is only a single hydrogen–hydrogen distance, which is used solely for discretization.

Let *n*_*H*_ and *n*_*H̅*_ denote the number of solutions found by the Branch-and-Prune (BP) algorithm using the *H* and *H̅* orderings, respectively. To quantify the effect of pruning induced by hydrogen-based interval distances, we evaluate the ratio *n*_*H*_/(*n*_*H*_ + *n*_*H̅*_) over a set of DDGP instances constructed with varying levels of uncertainty. Figure 4 shows box plots of this ratio for four interval lengths: δ = 0.2, 0.5, 1.0, 1.5 Å.

A value of 0.5 for this ratio indicates that the number of solutions obtained under both orderings is the same. Values substantially below 0.5 reveal a strong pruning effect attributable to the use of hydrogen–hydrogen interval constraints.

Across all four settings, the third quartile of the distribution remains below 0.2, implying that in at least 75% of the instances, the inclusion of hydrogen-based pruning distances results in a reduction of more than 75% in the number of feasible solutions. Furthermore, the median is consistently below 0.1, indicating that for at least half of the instances, over 88.9*%* of the solutions found by BP are pruned when the hydrogen–hydrogen constraint is removed.

Since the only difference between the *H* and *H̅* orderings lies in the use of hydrogen-to-hydrogen interval constraints, these results offer strong evidence that even imprecise, interval-based geometric information can be systematically leveraged to restrict the search space in protein loop modeling. In particular, short-range distances derived from NMR experiments can serve as effective pruning tools, improving the efficiency and selectivity of DDGP-based conformational search.

## Conclusions

In this work, we proposed a refinement of the Discretizable Distance Geometry Problem (DDGP) formulation for protein loop modeling by incorporating hydrogen atoms bonded to the protein backbone. This enhancement is particularly justified in the context of Nuclear Magnetic Resonance (NMR) data, where short-range distances between hydrogens can be experimentally measured and serve as geometric constraints.

The key contribution of this work lies in demonstrating that the inclusion of hydrogen atoms in the DDGP ordering, used as discretization and pruning elements, significantly reduces the search space of the problem. This effect was quantified through computational experiments involving loops of 4, 8, and 12 residues, where the Branch-and-Prune algorithm, when applied with our proposed hydrogen-enriched ordering, consistently generated fewer candidate structures compared to methods that ignore hydrogens.

These findings reinforce the potential of DDGP-based approaches to integrate experimentally accessible but uncertain data in a systematic way. The proposed ordering strategy provides a general and practical mechanism for incorporating hydrogen information into loop modeling pipelines, with benefits in both computational efficiency and biological plausibility of the resulting conformations.

Finally, we emphasize that this study follows the classical loop-closure setting, where the loop end points (or equivalently, selected *C*_α_ positions/distances anchoring the loop) are assumed to be fixed, as is customary in TLCP/LCP formulations. In practical prediction scenarios and in NMR-driven modeling, however, boundary atoms may also be mobile and only partially determined. Extending the present approach to account for partially mobile or uncertain boundaries (for instance, by incorporating additional restraints involving boundary atoms or by coupling loop closure with an outer refinement step) is a natural direction for future work. Another natural extension is to move beyond backbone (and backbone-bonded hydrogens) and incorporate side-chain geometry and restraints, so that additional experimentally accessible contacts can be exploited to further refine the conformational ensemble.

## Data Availability

The codes provided by the authors at https://github.com/romulomarques/bpl consist of the instances folder and the adaptation of the BP solver in Python. The complete computational results utilized in this paper are also available in the “tests” folders.

## References

[1] Shehu A., Kavraki L. E. (2012). Modeling structures and motions of loops in protein
molecules. Entropy.

[2] McHugh S., Rogers J., Solomon S., Yu H., Lin Y.-S. (2016). Computational
methods to design cyclic peptides. Curr. Opin.
Chem. Biol..

[3] Wang T. (2024). Comprehensive
assessment of protein loop modeling programs on large-scale
datasets: prediction accuracy and efficiency. Briefings Bioinf..

[4] Go̅ N., Scheraga H. A. (1970). Ring closure and
local conformational deformations
of chain molecules. Macromolecules.

[5] Wedemeyer W. J., Scheraga H. A. (1999). Exact Analytical Loop Closure in Proteins Using Polynomial
Equations. J. Comput. Chem..

[6] Coutsias E. F., Seok C., Jacobson M. P., Dill K. A. (2006). Resultants and loop
closure. Int. J. Quantum Chem..

[7] O’Donnell T., Agashe V., Cazals F. (2023). Geometric constraints within tripeptides
and the existence of tripeptide reconstructions. J. Comput. Chem..

[8] Porta J., Ros L., Thomas F., Torras C. (2005). A branch-and-prune solver for distance
constraints. IEEE Transactions on Robotics.

[9] Porta J., Ros L., Thomas F., Corcho F., Cantó J., Pérez J. (2007). Complete maps
of molecular-loop conformational spaces. J.
Comput. Chem..

[10] Sippl M., Scheraga H. A. (1986). Cayley-Menger coordinates. Proceedings
of the National Academy of Sciences USA.

[11] Neto L., Lavor C., Lodwick W. (2021). A note on
the Cayley-Menger determinant
and the molecular distance geometry problem. Inf. Sci..

[12] Coutsias E. F., Seok C., Jacobson M. P., Dill K. A. (2004). A kinematic view
of loop closure. J. Comput. Chem..

[13] Labiak R., Lavor C., Souza M. (2022). Distance geometry
and protein loop
modeling. J. Comput. Chem..

[14] Liberti L., Lavor C., Maculan N., Mucherino A. (2014). Euclidean
distance geometry and applications. SIAM Review.

[15] Wüthrich K. (1989). Protein structure
determination in solution by nuclear magnetic resonance spectroscopy. Science.

[16] Donald, B. Algorithms in Structural Molecular Biology; MIT Press: 2011.

[17] Lavor C., Liberti L., Maculan N., Mucherino A. (2012). The discretizable
molecular distance geometry problem. Computational
Optimization and Applications.

[18] Mucherino A., Lavor C., Liberti L. (2012). The discretizable distance
geometry
problem. Optimization Letters.

[19] Crippen, G. ; Havel, T. Distance Geometry and Molecular Conformation; Wiley: 1988.

[20] Gibson K. D., Scheraga H. A. (1997). Energy minimization of rigid-geometry polypeptides
with exactly closed disulfide loops. J. Comput.
Chem..

[21] Lavor C., Liberti L., Donald B., Worley B., Bardiaux B., Malliavin T., Nilges M. (2019). Minimal NMR distance information
for rigidity of protein graphs. Discrete Applied
Mathematics.

[22] Liberti L., Lavor C., Maculan N. (2008). A branch-and-prune algorithm for
the molecular distance geometry problem. International
Transactions in Operational Research.

[23] Cassioli A., Bordiaux B., Bouvier G., Mucherino A., Alves R., Liberti L., Nilges M., Lavor C., Malliavin T. (2015). An algorithm to enumerate all possible
protein conformations
verifying a set of distance constraints. BMC
Bioinf..

[24] Malliavin T., Mucherino A., Lavor C., Liberti L. (2019). Systematic exploration
of protein conformational space using a distance geometry approach. J. Chem. Inf. Model..

[25] Lavor C., Lee J., Lee-St John A., Liberti L., Mucherino A., Sviridenko M. (2012). Discretization
orders for distance geometry problems. Optim.
Lett..

[26] Cassioli A., Günlük O., Lavor C., Liberti L. (2015). Discretization vertex
orders in distance geometry. Discrete Applied
Mathematics.

[27] Maioli D. S., Lavor C., Gonçalves D. S. (2017). A note on computing the intersection
of spheres in *R*
^
*n*
^. ANZIAM J..

[28] Liberti L., Lavor C., Mucherino A., Maculan N. (2011). Molecular distance
geometry methods: from continuous to discrete. International Transactions in Operational Research.

[29] Billeter M., Braun W., Wüthrich K. (1982). Sequential
Resonance Assignments
in Protein H Nuclear Magnetic Resonance Spectra: Computation of Sterically
Allowed Proton-Proton Distances and Statistical Analysis of Proton-Proton
Distances in Single Crystal Protein Conformations. J. Mol. Biol..

[30] Rowland R. S., Taylor R. (1996). Intermolecular Nonbonded Contact
Distances in Organic
Crystal Structures: Comparison with Distances Expected from van der
Waals Radii. J. Phys. Chem..

[31] Lavor C., Liberti L., Mucherino A. (2013). The interval
Branch-and-Prune algorithm
for the discretizable molecular distance geometry problem with inexact
distances. Journal of Global Optimization.

[32] Lavor C., Souza M., Carvalho L. M., Gonçalves D. S. (2021). Improving the sampling process in the
interval Branch-and-Prune algorithm
for the discretizable molecular distance geometry problem. Appl. Math. Comput..

[33] Haynes, W. M. ; Lide, D. R. ; Bruno, T. J. CRC Handbook of Chemistry and Physics, 95th ed.; CRC Press: Boca Raton, 2014.

[34] Hou Y., Liu J., He J., Peng X., Niemi A. J. (2019). Study of correlations
between protein peptide plane dynamics and side chain dynamics. PLoS One.

